# The potential role of long-acting injectable cabotegravir–rilpivirine in the treatment of HIV in sub-Saharan Africa: a modelling analysis

**DOI:** 10.1016/S2214-109X(21)00025-5

**Published:** 2021-03-23

**Authors:** Andrew N Phillips, Loveleen Bansi-Matharu, Valentina Cambiano, Peter Ehrenkranz, Celicia Serenata, Francois Venter, Sarah Pett, Charles Flexner, Andreas Jahn, Paul Revill, Geoff P Garnett

**Affiliations:** a Institute for Global Health, University College London, London, UK; b Institute of Clinical Trials and Methodology, University College London, London, UK; c Bill & Melinda Gates Foundation, Seattle, WA, USA; d Ezintsha, Faculty of Health Sciences, University of the Witwatersrand, Johannesburg, South Africa; e School of Medicine and Bloomberg School of Public Health, Johns Hopkins University, Baltimore, MD, USA; f Ministry of Health, Lilongwe, Malawi; g International Training and Education Center for Health, Department of Global Health, University of Washington, Seattle, WA, USA; h Centre for Health Economics, University of York, York, UK

## Abstract

**Background:**

The use of a combination of the integrase inhibitor, cabotegravir, and the non-nucleoside reverse transcriptase inhibitor, rilpivirine, in a long-acting injectable form is being considered as an antiretroviral treatment option for people with HIV in sub-Saharan Africa. We aimed to model the effects of injectable cabotegravir–rilpivirine to help to inform its potential effectiveness and cost-effectiveness under different possible policies for its introduction.

**Methods:**

We used an existing individual-based model of HIV to predict the effects of introducing monthly injections of cabotegravir–rilpivirine for people with HIV in low-income settings in sub-Saharan Africa. We evaluated policies in the context of 1000 setting scenarios that reflected characteristics of HIV epidemics and programmes in sub-Saharan Africa. We compared three policies for introduction of injectable cabotegravir–rilpivirine with continued use of dolutegravir-based oral regimens for: all individuals on antiretroviral therapy (ART); individuals with a recently measured viral load of more than 1000 copies per mL (signifying poor adherence to oral drugs, and often associated with drug resistance); and individuals with a recently measured viral load of less than 1000 copies per mL (a group with a lower prevalence of pre-existing drug resistance). We also did cost-effectiveness analysis, taking a health system perspective over a 10 year period, with 3% discounting of disability-adjusted life-years (DALYs) and costs. A cost-effectiveness threshold of US$500 per DALY averted was used to establish if a policy was cost-effective.

**Findings:**

In our model, all policies involving the introduction of injectable cabotegravir–rilpivirine were predicted to lead to an increased proportion of people with HIV on ART, increased viral load suppression, and decreased AIDS-related mortality, with lesser benefits in people with a recently measured viral load of less than 1000 copies per mL. Its introduction is also predicted to lead to increases in resistance to integrase inhibitors and non-nucleoside reverse transcriptase inhibitors if introduced in all people with HIV on ART or in those with a recently measured viral load of less than 1000 copies per mL, but to a lesser extent if introduced in people with more than 1000 copies per mL due to concentration of its use in people less adherent to oral therapy. Consistent with the effect on AIDS-related mortality, all approaches to the introduction of injectable cabotegravir–rilpivirine are predicted to avert DALYs. Assuming a cost of $120 per person per year, use of this regimen in people with a recently measured viral load of more than 1000 copies per mL was borderline cost-effective (median cost per DALY averted across setting scenarios $404). The other approaches considered for its use are unlikely to be cost-effective unless the cost per year of injectable cabotegravir–rilpivirine is considerably reduced.

**Interpretation:**

Our modelling suggests that injectable cabotegravir–rilpivirine offers potential benefits; however, to be a cost-effective option, its introduction might need to be carefully targeted to individuals with HIV who might otherwise have suboptimal adherence to ART. As data accumulate from trials and implementation studies, such findings can be incorporated into the model to better inform on the full consequences of policy alternatives.

**Funding:**

Bill & Melinda Gates Foundation, including through the HIV Modelling Consortium (OPP1191655).

## Introduction

Until 2020, antiretroviral drug regimens in sub-Saharan Africa consisted of two nucleoside reverse transcriptase inhibitors (most commonly lamivudine and tenofovir disoproxil fumarate) and a non-nucleoside reverse transcriptase inhibitor (most commonly efavirenz). Due to concerns over increasing transmission of resistance to non-nucleoside reverse transcriptase inhibitors, in 2019, WHO strongly recommended a change in regimen for people initiating first-line antiretroviral therapy (ART) to the integrase inhibitor, dolutegravir, with lamivudine and tenofovir disoproxil fumarate.^1^ To date, only oral drug regimens have been available.

A combination of the integrase strand transfer inhibitor, cabotegravir, and the non-nucleoside reverse transcriptase inhibitor, rilpivirine, is likely to become available as ART in long-acting injectable form, with one or two monthly intramuscular injections.^2^, ^3^, ^4^ There is interest in the possibility of injectable cabotegravir–rilpivirine use in sub-Saharan Africa.^5^ Potential advantages of injectable forms over current oral regimens are that injections result in adequate drug concentrations for a 1–2 month period, without dependence on adherence to daily oral pill taking. Disadvantages include cabotegravir appearing to have a lower barrier to resistance than does dolutegravir;^6^ a long so-called tail of cabotegravir drug concentrations if combination treatment is stopped, which might lead to selection of drug resistance if other antiretroviral drugs are not taken, potentially risking class-wide integrase strand transfer inhibitor resistance;^7^ injections might be painful and induce injection-site skin reactions; and the possibility of higher costs with injectables forms than with oral therapy for procurement and delivery, as well as a greater time commitment for clinic staff. Studies of implementation of injectable cabotegravir–rilpivirine in low-income settings in sub-Saharan Africa might be guided usefully by modelling exercises exploring potential effects in different subpopulations. We aimed to model the effects of injectable cabotegravir–rilpivirine on ART outcomes in patients with HIV from these settings and to assess its cost-effectiveness to help to inform potential policies for its introduction.

**Research in context**

**Evidence before this study**

The use of a combination of the integrase inhibitor, cabotegravir, and the non-nucleoside reverse transcriptase inhibitor, rilpivirine, in long-acting injectable form as an antiretroviral treatment option in sub-Saharan Africa is being considered. Modelling can help to inform potential policies for its introduction. We searched Web of Science, with no restrictions on language, using the search terms “cabotegravir” AND (list of countries in sub-Saharan Africa) on June 30, 2020, and identified one modelling study that has considered the cost-effectiveness of the introduction of such treatment in adolescents in the context of Kenya, and found that this could be cost-effective if associated with an additional annual cost of US$89 or less.

**Added value of this study**

We used an existing individual-based model of HIV to consider the predicted effects and cost-effectiveness of the introduction of monthly injectable cabotegravir–rilpivirine. We considered drug resistance to cabotegravir and rilpivirine, cross resistance with oral drugs of the same classes, and the long tail of cabotegravir drug concentrations in people who stop attending for monthly injections.

**Implications of all the available evidence**

Injectable cabotegravir–rilpivirine offers potential benefits; however, its introduction needs to be carefully targeted to individuals who might otherwise have suboptimal adherence to ART and studied in pilot implementation projects to ascertain if it is likely to be a cost-effective option.

## Methods

### Modelling approach

The HIV synthesis model is an individual-based simulation model that has been described in detail elsewhere.^8^, ^9^ Each time the model programme is run, it creates a simulated dataset of attributes of a population of adults from 1989 (the notional start date of the HIV epidemic), with updates every month (adapted from 3 months for this exercise) on variables including age, sex, presence of a primary condomless sex partner and number of short-term condomless sex partners, HIV testing, male circumcision, use of pre-exposure prophylaxis and, for HIV-positive people, time from infection, CD4 cell count, viral load, specific antiretroviral drugs being used, antiretroviral drug adherence (for oral drugs), and specific drug resistance mutations. In this analysis, adherence refers to the extent of optimal pill taking as prescribed in the 1 month period among people who are attending clinic. ART interruption is modelled separately. Adherence to the specific drugs being taken and the presence of drug resistance mutations jointly determine the current amounts of suppressive antiviral effect of the regimen at any point in time. Through sampling of several parameter values, we create a series of so-called setting scenarios reflecting uncertainty in assumptions and a range of characteristics similar to those seen in southern and east Africa. For example, we sample parameters relating to rates of HIV testing; linkage and retention; adherence to oral antiretroviral drugs; resistance emergence, transmission, and persistence; ART interruption; extent of implementation of viral load monitoring; and rate of switching to second-line therapy after detected virological failure. A table of parameters and the criteria for a setting scenario to be accepted are provided in the appendix (pp 2–10). With our setting scenarios, we aim to reflect subsettings within countries, not just countries in aggregate.

For each setting scenario, we randomly sampled injectable cabotegravir–rilpivirine introduction policies, modelling transition to injectable cabotegravir–rilpivirine for three groups: first, all people on ART; second, people with a most recently measured viral load of more than 1000 copies per mL (as a means of selecting people with a tendency for poorer adherence to oral drugs); and third, people with a recently measured viral load of less than 1000 copies per mL (a group with a lower prevalence of pre-existing drug resistance).

### Context

We assumed a context where dolutegravir was introduced over 2019–20 and where all people on ART were switched to tenofovir disoproxil fumarate–lamivudine–dolutegravir. If there were two viral load values above 1000 copies per mL on this regimen, it was switched to boosted protease inhibitor plus zidovudine and lamivudine. We further assumed that viral load monitoring was in place, but with varying levels of implementation across setting scenarios.

### Cost-effectiveness analysis

Outcomes compared include the proportion of people on ART with viral suppression, AIDS-related deaths, disability-adjusted life-years (DALYs), and net DALYs (which account for costs of a policy on the basis of a cost-effectiveness threshold of US$500 per DALY averted). The cost-effectiveness threshold should reflect the health gains that could have been delivered through other interventions that can no longer be provided if the evaluated interventions are funded (ie, the health opportunity costs).^10^ HIV care in sub-Saharan Africa is mainly funded by external development assistance; therefore, this is unlikely to depend as much on a country's income level for HIV spending as for general health care. Although uncertain, $500 averted per DALY is likely to be at the upper end for lower-income countries in the region, on the basis of evidence concerning how resources could otherwise be used. Incremental cost-effectiveness ratios for funded interventions such as the expansion of ART to every patient with diagnosed HIV, which includes an increased need for testing and use of viral load monitoring, are around this value.^11^, ^12^ Given the discussions around feasibility of implementation at the time of adoption, these appear to be on the borderline of cost-effective interventions. Furthermore, for South Africa, a middle-income country that funds most of its programme through domestic funding, a cost-effectiveness threshold of $750 has been proposed because this is approximately the cost per life-year averted of HIV interventions at the borderline for inclusion within the South African HIV Investment Case, which prioritises use of the national HIV budget on the basis of intervention cost-effectiveness.^13^

The cost of tenofovir disoproxil fumarate–lamivudine–dolutegravir, including the supply chain, is assumed to be $78 per patient per year.^14^ The cost at which injectable cabotegravir–rilpivirine could be delivered is unknown and we initially use a cost of $120 per patient per year for illustrative purposes only, which includes all costs associated with its delivery, including that of the drug and supply chain, and any additional costs in the clinic beyond those required in the context of current oral drug use. Costs of providing clinic visits in the context of current oral drug use ($80 per patient per year unless viral load is known to be suppressed, in which case $40 per year; we make no explicit assumptions on prescription lengths for tenofovir disoproxil fumarate–lamivudine–dolutegravir) and viral load testing ($22 per test) are considered separately from these costs. Absolute numbers of health-related events, costs, and DALYs are relevant for a population with a size of approximately 10 million adults in 2020. The cost-effectiveness analysis was done from a health-care perspective, with costs and health outcomes both discounted to present $ values at 3% per annum. Other unit costs and disability weights are described in the appendix (pp 11–12).

### Modelling drug resistance

Before explaining how we modelled injectable cabotegravir–rilpivirine, we first describe how we modelled the effects of oral drug regimens. For an individual on oral ART in the model, the risk of new drug resistance mutations arising depends on their adherence in the current month, which is modelled as a percentage between 0% and 100% full adherence. People with an adherence of less than 80% in a given month have a substantially higher risk of drug resistance than those with an adherence of 80% or above. Risk of resistance mutations is drug-specific, but the relative rate of resistance between individuals with at least 80% adherence and those with less than 80% adherence is assumed to be the same across drugs. These assumptions have been previously shown to lead to levels of drug resistance consistent with observed data.^8^, ^15^ People have a long-term average tendency to adhere to oral drugs, but there is variability within individuals by month.^8^ The current amount of activity of the overall regimen is the sum of the activity of each drug in the regimen, accounting for the intrinsic drug potency and the current level of resistance to the drug, in which the potency conveys the contribution of a drug to the antiviral activity of a regimen against a virus with no resistance, informed by early short-term studies of drugs in development used as monotherapy and inferred from the potency of regimens of drug combinations.^8^, ^15^ People can interrupt oral drugs and, if so, there is a high probability that this coincides with a stop in clinic attendance. In people with low long-term tendency to adhere to ART, there is an increased rate with which such interruption can occur. With the inclusion of long-acting injectable drugs, we generalised the adherence percentage to be thought of as a measure of the degree to which concentrations of the drugs being taken are optimal in the 1 month period. Where the percentage is above 80%, we referred to this as indicating optimal drug concentrations for the month; where it is below 80%, we referred to such as indicating suboptimal drug concentrations. We assumed that injections are monthly.

We modelled indicative non-nucleoside reverse transcriptase inhibitor mutations at reverse transcriptase codon positions 101, 103, 138, 181, 188, and 190. The risk of resistance mutations with rilpivirine is higher than with efavirenz.^16^ We accounted for the fact that there is cross resistance between efavirenz and rilpivirine, although rilpivirine remains active in the presence of the Lys103Asn mutation. The risk of integrase mutations (Gln148 or Arg263 are the major integrase inhibitor mutations considered) is two times higher with cabotegravir than with dolutegravir. There is extensive cross resistance between cabotegravir and dolutegravir. The potency of each drug (the activity amount if no resistance mutations are present) is 1·0 for all nucleoside reverse transcriptase inhibitors. Efavirenz, rilpivirine, dolutegravir, and cabotegravir are assumed to have the same potency and this is set to 1·5 or 2·0 (determined by sampling for each run), and 2·0 for atazanavir and lopinavir.

Cabotegravir has a long half-life and, if no further injections or oral drugs are taken, the cabotegravir–rilpivirine concentration after the most recent injection is assumed to remain above 80% of optimal drug concentration for 2 months (ie, at low-resistance risk) and then fall to a suboptimal concentration (ie, at high-resistance risk) for the following 3, 6, or 12 months. The suboptimal concentration period of 3, 6, or 12 months is variable across individuals. During this period, there is some antiviral effect of the regimen. Thereafter, the drug concentration effectively falls to 0 and there is no residual antiviral effect or risk of resistance development.

Variations in the assumptions explored (with the percentage of setting scenarios reflecting our uncertainty) included a two times higher (33% of setting scenarios) or two times lower (33% of setting scenarios) rate of ART interruption with injectable cabotegravir–rilpivirine compared with oral antiretrovirals, rather than the two being the same (33% of setting scenarios). Additionally, people with lower adherence might tend to correspond to individuals with lower levels of sexual transmission risk (20% of setting scenarios).

### Role of the funding source

The programme officers for the funder (GPG and PE) had input into the modelling design and are coauthors on this report as a result of their input and expertise. The funder of the study had no other role in study design, data collection, data analysis, data interpretation, or writing of the report.

## Results

Table 1 shows the characteristics of the population in July, 2020, across 1000 setting scenarios, with observed values from sub-Saharan African countries provided for context, although we note that we aimed for our setting scenarios to also reflect the range of epidemic characteristics in specific subsettings within countries.

**Table 1:** Characteristics of HIV epidemics and programmes in 1000 setting scenarios at baseline (2020)

	**Sampled value**	**Examples of observed data**
HIV prevalence (aged 15–49 years), %	17% (5–33)	Zimbabwe 2016 13%, Tanzania 2017 5%, Uganda 2017 6%, Lesotho 2017 24%, Eswatini 2017 27%, Malawi 2016 10%, Namibia 2017 12%, Zambia 2016 11%, Cameroon 2017 3·4%, Côte d'Ivoire 2017–18 2·5%
HIV incidence (aged 15–49 years), per 100 person-years	1·10 (0·25–2·87)	Malawi 2016 0·37, Zambia 2016 0·66, Zimbabwe 2016 0·45, Lesotho 2017 1·55, Namibia 2016 0·40, Eswatini 2017 1·48, Tanzania 2017 0·27, Cameroon 2017 0·27
Proportion of HIV-positive people diagnosed, %	88% (69–95)	Malawi 2016 77%, Zambia 2016 67%, Zimbabwe 2016 74%, Namibia 2017 86%, Tanzania 2017 52%, Ethiopia 2018 72%, Côte d'Ivoire 2017/18 37%, Cameroon 2017 47%
Proportion of diagnosed HIV-positive people on ART, %	88% (72–97)	Lesotho 2016–17 92%, South Africa 2017 71%, Eswatini 2016–17 87%, Namibia 2017 96%, Zambia 2016 87%, Tanzania 2016–17 94%, Ethiopia 2017–18 99%, Malawi 2016 91%, Uganda 2016–17 90%, Cameroon 2017 91%, Zimbabwe 2016 87%, Côte d'Ivoire 2017–18 88%, Cameroon 2017 91%
Proportion of all HIV-positive people with a viral load of <1000 copies per mL, %	64% (44–80)	Zambia 2016 60%, Malawi 2016 68%, Zimbabwe 2016 60%, Eswatini 2017 73%, Lesotho 2017 68%, Tanzania 2017 52%, Uganda 2017 60%, Namibia 2017 77%, Ethiopia 2018 70%, Côte d'Ivoire 2017–18 40%, Cameroon 2017 47%
Proportion of ART-experienced people who have started second-line ART, %	14% (6–35)	Malawi approximately 3%*
Proportion of people on ART who have a viral load of <1000 copies per mL, %	90% (74–96)	Zambia 2016 88% (men) and 90% (women), Malawi 2016 90% (men) and 92% (women), Zimbabwe 2016 84% (men) and 88% (women), Namibia 2017 92% (men) and 90% (women), Tanzania 2017 89% (men) and 83% (women), Ethiopia 2018 95% (men) and 87% (women), Côte d'Ivoire 2017–18 76%, Cameroon 2017 80%
Proportion of people on ART who have a CD4 count of <500 cells per μL, %	53% (49–60)	Eswatini 2016–17 40%, Malawi 2016 52%, Tanzania 2017–18 55%, Zambia 2016 59%
Proportion of ART-naive people initiating ART who have non-nucleoside reverse transcriptase inhibitor resistance, %	13% (2–35)	Angola 2012 14%, Botswana 2016 8%, South Africa 2017 14%, Zimbabwe 2015 10%, Namibia 9%, Uganda 2016 16%, Cameroon 8%†

Data are median (90% range), unless otherwise indicated. ART=antiretroviral therapy.

* According to quarterly reports by Malawi's Ministry of Health.

† According to WHO's HIV drug resistance report 2017.

Table 2 shows the predicted effects of the introduction of injectable cabotegravir–rilpivirine for the three groups (ie, introduction targeted at all patients on ART, only at those with high viral loads, and only at those with low viral loads). If injectable cabotegravir–rilpivirine use was dependent on a recently measured viral load of more than 1000 copies per mL, patients would have a high prevalence of resistant virus when starting the regimen (table 2). All policies involving the introduction of injectable cabotegravir–rilpivirine were predicted to lead to an increase in the proportion of people on ART who have viral load suppression and a decrease in AIDS-related deaths, compared with no introduction. Its introduction was also predicted to lead to increases in integrase inhibitor and non-nucleoside reverse transcriptase inhibitor resistance, if introduced in every patient on ART or in individuals with a recently measured viral load of less than 1000 copies per mL, but not in those with viral loads of more than 1000 copies per mL. This effect is due to the fact that use of injectable cabotegravir–rilpivirine is concentrated among people with reduced adherence to oral therapy, who would carry a risk of integrase inhibitor resistance with oral therapy. Consistent with the effect on AIDS-related mortality, all approaches to the introduction of injectable cabotegravir–rilpivirine were predicted to avert DALYs.

**Table 2:** Predicted effects of three implementation approaches for the introduction of injectable CTG–RPV

		**Implementation in all people on ART (n=333 setting scenarios)**	**Implementation in people with a recently measured viral load of >1000 copies per mL (n=334 setting scenarios)**	**Implementation in people with a recently measured viral load of <1000 copies per mL (n=333 setting scenarios)**
Percentage of people on ART on injectable CTG–RPV, %		99% (99 to 99; 97 to 100)	26% (25 to 27; 13 to 44)	86% (86 to 87; 75 to 94)
People starting injectable CTG–RPV				
	Percentage with viral load >1000 copies per mL*, %	40% (39 to 41; 26 to 56)	69% (68 to 71; 52 to 81)	4% (3 to 4; 1 to 7)
	Percentage for whom first-line ART failed†, %	13% (0·12 to 0·13; 0·05 to 0·23)	25% (24 to 26; 13 to 38)	7% (6 to 7; 2 to 18)
	Percentage with integrase inhibitor resistance, %	2% (2 to 2; 0 to 4)	7% (6 to 7; 2 to 13)	1% (0·01 to 0·02; 0·00 to 0·04)
	Percentage with non-nucleoside reverse transcriptase inhibitor resistance, %	24% (23 to 26; 11 to 46)	38% (37 to 40; 18 to 59)	18% (17 to 20; 7 to 39)
	Percentage male, %	40% (40 to 41; 34 to 48)	46% (45 to 47; 38 to 55)	39% (39 to 40; 33 to 46)
	Percentage aged <25 years, %	5% (5 to 6; 1 to 12)	3% (3 to 4; 1 to 8)	5% (4 to 5; 1 to 10)
Introduction of injectable CTG–RPV *vs* no introduction				
	Difference in proportion of people with suboptimal drug concentrations (ie, <80% adherence to oral therapy) among people who started injectable cabotegravir–rilpivirine, %	+8·0% (7·1 to 8·8; 0·0 to 19·5)	+11·7% (10·3 to 2·6; 0·0 to 26·8)	+7·4% (6·5 to 8·3; 1·2 to 18·0)
	Difference in proportion of people with a viral load of <1000 copies per mL among people on ART, %	+5·3% (4·8 to 5·7; 1·0 to 14·4)	+4·1% (3·7 to 4·4; 0·7 to 11·2)	+3·0% (2·7 to 3·3; 0·4 to 8·5)
	Difference in AIDS-related mortality among people with HIV, per 100 person-years	−0·19 (−0·21 to −0·16; −0·68 to 0·08)	−0·17 (−0·19 to −0·15; −0·54 to −0·01)	−0·05 (−0·06 to −0·04; −0·28 to 0·11)
	Difference in proportion of people with HIV with integrase inhibitor resistance, %	+0·8% (0·5 to 1·2; −3·0 to 6·0)	−0·4% (−0·5 to −0·3; −2·1 to 0·9)	+1·0% (0·7 to 1·2; −2·0 to 4·7)
	Difference in proportion of people with HIV with non-nucleoside reverse transcriptase inhibitor resistance, %	+4·3% (4·1 to 4·5; 1·9 to 7·7)	+1·5% (1·3 to 1·6; 0·2 to 3·8)	+3·4% (3·3 to 3·6; 1·5 to 6·2)
	Difference in HIV incidence in people aged 15–49 years, per 100 person-years	−0·04 (−0·05 to −0·03; −0·14 to 0·04)	−0·02 (−0·02 to −0·01; −0·10 to 0·06)	−0·02 (−0·03 to −0·01; −0·10 to 0·04)
	DALYs averted, per year over 10 years	30 400 (27 300 to 33 400; 400 to 91 800)	17 900 (15 700 to 20 000; −3200 to 54 800)	16 700 (15 000 to 18 500; −2800 to 47 600)
	Difference in cost, US$ million per year over 10 years (with discounting at 3% per annum)	+42·9 (40·2 to 45·6; 9·0 to 86·5)	+5·4 (4·6 to 6·2; −4·9 to 18·7)	+42·5 (39·8 to 45·1; 10·1 to 83·9)
	Net DALYs averted, per year over 10 years‡	−55 500 (−60 400 to −50 500; −135 000 to 7100)	7100 (4700 to 9600);–18 000 to 54 800)	−68 200 (−72 900 to −63 500; −143 600 to −10 700)
	Median cost per DALY averted (90% range)	$1638 (390 to 59 524)	$404 (dominant to dominated)	$2808 (794 to dominated)
	Difference in proportion of setting scenarios in which injectable CTG–RPV is cost-effective, % (95% CI)	7% (5 to 11)	59% (53 to 64)	2% (0 to 3)

Data are mean (95% CI; 90% range) over 10 years, unless otherwise indicated. Percentage values are absolute (ie, percentage with CTG–RPV − percentage without CTG–RPV). ART=antiretroviral therapy. CTG–RPV=cabotegravir–rilpivirine. DALY=disability-adjusted life-year.

* Proportion of all initiations of injectable CTG–RPV over the 10 year period for which the most recent viral load was >1000 copies per mL, as opposed to the calculation of this proportion for each 3 month period in the 10 years and then taking the mean. This is important for this regimen in all people on ART because there are many initiations of injectable CTG–RPV in the first period (when the proportion of people with a most recent viral load of >1000 copies per mL is low), and few subsequent initiations that are all in people initiating ART for the first time (hence, viral load is >1000 copies per mL in almost all cases). Because we model true viral load, this is the actual viral load of the person and is not necessarily measured, whereas a clinic would only know a person's viral load if it was measured.

† These patients fulfilled the first-line failure or switch criteria.

‡ Difference in DALYs + (difference in costs/cost-effectiveness threshold).

With our assumed indicative cost of $120 per person per year for injectable cabotegravir–rilpivirine, overall programme costs were predicted to increase substantially with the policy for every patient on ART or for individuals with a viral load of less than 1000 copies per mL, but to a much lesser extent with the policy for those with a viral load of more than 1000 copies per mL (table 2). This difference is due to the targeting to people who are more costly to manage because of, for example, the high costs of second-line protease inhibitor-based regimens. Net DALYs are averted with the injectable cabotegravir–rilpivirine policy for those with a viral load of more than 1000 copies per mL. The introduction of such a regimen for this patient group was cost-effective in 59% (95% CI 53–64) of setting scenarios (table 3). Other policies for the introduction of injectable cabotegravir–rilpivirine were not cost-effective. These findings are based on our assumed cost of $120 for this regimen per person per year. We also asked the question of what the maximum cost of delivering injectable cabotegravir–rilpivirine would be for the introduction of the regimen's policy for individuals with a viral load of more than 1000 copies per mL to be cost-effective (ie, for the mean net DALY averted to be ≥0). We found such cost to be $131 per person per year.

**Table 3:** Sensitivity analyses showing percentage of setting scenarios in which injectable cabotegravir–rilpivirine is cost-effective in people with a recently measured viral load of >1000 copies per mL

		**Percentage of cost-effective setting scenarios**
Overall		59% (53–64)
Time cabotegravir is at suboptimal concentrations after last injection		
	3 months in 70% of people, 6 months in 20%, 1 year in 10%	64% (54–73)
	3 months in 33% of people, 6 months in 33%, 1 year in 33%	53% (44–62)
	3 months in 10% of people, 6 months in 20%, 1 year in 70%	60% (50–69)
Rate of antiretroviral interruption or restart with injectable CTG–RPV compared with oral antiretrovirals		
	Two times higher interruption or two times lower restart rate	61% (51–70)
	Equal interruption and restart rates	60% (50–69)
	Two times lower interruption or two times higher restart rate	55% (46–65)
People with lower adherence have a lower risk of sexual transmission (ie, fewer condomless partners)		
	Yes	55% (43–67)
	No	60% (53–65)
Percentage of people on ART who have a viral load of <1000 copies per mL at baseline		
	Highest tertile (>92%)	31% (23–40)
	Lowest tertile (<87%)	91% (83–95)
Percentage of all people with HIV who have a viral load of <1000 copies per mL at baseline*		
	Highest tertile (>70%)	45% (36–55)
	Lowest tertile (<59%)	69% (60–78)
Percentage of people on ART who are on a second-line (protease inhibitor-based) regimen at baseline		
	Highest tertile (>17%)	81% (72–87)
	Lowest tertile (<12%)	39% (30–48)

Data are % (95% CI). Baseline refers to 2020. Effects are compared with those of not introducing injectable CTG–RPV, according to characteristics of setting scenarios and additional changes in assumptions. ART=antiretroviral therapy. CTG–RPV=cabotegravir–rilpivirine.

* As a measure of overall scope of, and engagement with, the ART programme.

In further analyses (based on the cost of $120 per person per year for injectable cabotegravir–rilpivirine), we considered the effects of restricting setting scenarios in various ways on the percentage of scenarios in which a policy for the regimen in people with a recently measured viral load of more than 1000 copies per mL is cost-effective (table 3).

## Discussion

We did an exploratory modelling analysis to inform potential ways in which the introduction of injectable cabotegravir–rilpivirine could have a beneficial effect on ART outcomes in patients with HIV in sub-Saharan Africa. We found that this regimen could have a substantial positive effect if targeted to patients with a viral load of more than 1000 copies per mL (despite being on a tenofovir disoproxil fumarate–lamivudine–dolutegravir regimen), a group for whom adherence to pill taking is more likely to be suboptimal. Delivered at a cost of below $131 per person per year, injectable cabotegravir–rilpivirine is potentially cost-effective when used in this group. In individuals with a viral load of more than 1000 copies per mL on a tenofovir disoproxil fumarate–lamivudine–dolutegravir regimen, there is likely to be a considerable risk of integrase inhibitor resistance. Use of injectable cabotegravir–rilpivirine instead of the oral regimen is not predicted to result in any higher risk. Although increasing viral load suppression and reducing DALYs, the increased overall costs associated with use of injectable cabotegravir–rilpivirine in every patient on ART or in those with a viral load of less than 1000 copies per mL outweigh the benefits under our assumptions.

We note that the consequences of increased implementation complexity (including cold chain, syringes, coadministration of two separate products, the possible need for an oral cabotegravir–rilpivirine dose lead-in to rule out serious toxicity, a need to rule out hepatitis B before stopping tenofovir, a need to move away from injectable cabotegravir–rilpivirine during treatment for active tuberculosis, the burden on the patient of monthly clinic visits) and time inputs in clinics required with the monthly delivery of injectable cabotegravir–rilpivirine are not fully known yet. On the basis of our current assumptions, the cost associated with these factors would need to be considered within the proposed $131 per person per year ceiling cost. Injections once every 2 months rather than once every month partially reduces some of these costs. Additionally, some programmes are not able to carry out monitoring of viral load annually as most guidelines propose; therefore, this limits the ability to implement the policy we modelled.

Subpopulations that might be most susceptible to chronic poor adherence to daily oral drugs include adolescents and young adults (aged <20 years), and mobile workers who might have a particular aversion to carrying pills. Individuals who might benefit the most might also be those with highest sexual risk behaviour. These patients might be the subpopulations in whom injectable cabotegravir–rilpivirine is first introduced. We considered targeting injectable cabotegravir–rilpivirine according to recent viral load; however, implementation studies might consider other direct measures of adherence, such as measurement of drug concentrations.

We are mindful that selection of people based on increased viral load means that we are likely to select a group among whom levels of drug resistance are high, and our proposed policy does not include any drug resistance testing to detect this given that, until now, such testing has not been feasible for programmes. Our modelling attempts to take this into account and the predicted benefits of the policy are in this context. However, such a policy might be viewed as a clinically unacceptable approach nevertheless.

Given that a high proportion of people might consider injectable cabotegravir–rilpivirine as a convenient option, a policy of introducing the regimen only in patients with a viral load of more than 1000 copies per mL could lead to an incentive of poor adherence to oral drugs in individuals who wish to access the injectable form; therefore, the approach to implementation would have to be carefully considered. Because a person with close to 100% adherence to oral drugs is likely to have a lower risk of resistance with tenofovir disoproxil fumarate–lamivudine–dolutegravir than with injectable cabotegravir–rilpivirine, if given this information by clinical staff, patients could effectively self-select to use injectable cabotegravir–rilpivirine if they have concerns over their ability to sustain adherence to oral drugs. By contrast, those patients who are confident in their ability might prefer the reassurance of a predicted very low risk of resistance development. In practice, the approach tested for provision of injectable cabotegravir–rilpivirine might be based on allowing people to make an informed choice. It will be important to run such pilot programmes to see how use plays out. Our modelling results imply that such an approach is unlikely to be cost-effective at the drug costs and dosing regimen considered; however, uncertainty remains and our modelling should be updated when data on actual programme experience are available.

We considered whether our conclusions were affected by plausible variations between setting scenarios or by uncertainty over assumptions. Most notably, we found that the policy was less likely to be cost-effective in the context of programmes in which the proportion of people on ART who had a viral load of less than 1000 copies per mL was higher. We also found that the policy was more likely to be cost-effective in settings in which there was a higher proportion of people on second-line ART. This finding probably reflects that, due to the high cost of second-line regimens, the benefits provided by injectable cabotegravir–rilpivirine in achieving higher levels of viral suppression are greater.

A 2020 modelling study considered the minimum additional cost of injectable cabotegravir–rilpivirine, compared with oral therapy, for this regimen to be considered a cost-effective option in adolescents in Kenya.^17^ Using the same $500 per DALY averted cost-effectiveness threshold as in our study, the authors found that an additional annual cost of up to $89 beyond the cost of oral treatment could be possible. Although in a somewhat different context, this cost is not far from the equivalent figure of $53 for our study ($131 – $78 for tenofovir disoproxil fumarate–lamivudine–dolutegravir).

Modelling can help to combine the pieces of information available, making assumptions as necessary and exploring the effect of these assumptions. This approach helps to establish what the potential effects of policy options and their cost-effectiveness could be, but it does not provide empirical data in the way that a randomised trial does, and it is important that this difference is understood. The main limitation of this work is uncertainty over many of the assumptions, particularly related to injectable cabotegravir–rilpivirine, because it is yet to be introduced into clinical practice.

In conclusion, our modelling suggests that injectable cabotegravir–rilpivirine offers potential benefits to patients with HIV on ART in low-income settings in sub-Saharan Africa; however, its introduction would probably need to be carefully targeted if it is to offer a cost-effective option, and much uncertainty remains. As data accumulate from trials and implementation studies, such findings can be incorporated into the model to better inform on the full consequences of policy alternatives.

## Data sharing

The model programme is available on figshare.

## Declaration of interests

CWF reports personal fees from Merck, Mylan Pharmaceuticals, ViiV Healthcare, Gilead Sciences, and Algernon Pharmaceuticals, outside of the submitted work. CWF also has an issued patent for semi-solid prodrug solid drug nanoparticles and for anhydrous nanoprecipitation for the preparation of nanodispersions of tenofovir disoproxil fumarate in oils as candidate long-acting injectable depot formulations. SP reports grants from Gilead Sciences, ViiV Healthcare, Janssen-Cilag, and European and Developing Countries Clinical Trials; and salary support from Medical Research Council grant (MC_UU_12023/23) (MC_UU_12023/26), outside of the submitted work. FV reports grants from US Agency for International Development, Unitaid, Aidsfonds, South African Medical Research Council, National Institutes of Health, ViiV, and Merck; non-financial support from Gilead and ViiV; and personal fees from Gilead, ViiV, Mylan, Merck, Adcock-Ingram, Aspen, Abbott, Roche, Johnson & Johnson, and Virology Education, outside of the submitted work. PE and GPG were programme officers for the funder and had input into the modelling design. All other authors declare no competing interests. This Article is based on research funded by the Bill & Melinda Gates Foundation. The findings and conclusions contained within are those of the authors and do not necessarily reflect the official positions or policies of the Bill & Melinda Gates Foundation.
