# Supplementary material for: The potential role of long-acting injectable cabotegravir–rilpivirine in the treatment of HIV in sub-Saharan Africa: a modelling analysis
Source: Lancet Glob Health. 2021 Mar 23;9(5):e620–7. doi: 10.1016/S2214-109X(21)00025-5 (PMC8050198; doi:10.1016/S2214-109X(21)00025-5)
Supplement: Supplementary appendix [file mmc1.pdf]

THE LANCET
Global Health

Supplementary appendix

This appendix formed part of the original submission and has been peer reviewed. We post it as supplied by the authors.

Supplement to: Phillips AN, Bansi-Matharu L, Cambiano V, et al. The potential role of long-acting injectable cabotegravir–rilpivirine in the treatment of HIV in sub-Saharan Africa: a modelling analysis. *Lancet Glob Health* 2021; published online March 23. http://dx.doi.org/10.1016/S2214-109X(21)00025-5.

The potential role for long-acting injectable cabotegravir / rilpivirine in treatment of HIV in sub-Saharan Africa: a modelling analysis

Appendix

Table S1. Parameter distributions sampled for each model run together with the distribution for the 500 setting-scenarios. Details of the model are described previously (1, 2). Setting scenarios were selected to be part of the 500 if they fulfilled the following criteria in 2020: prevalence (age 15-49) > 1% and < 35%; ratio of prevalence in 25-29 year old women to prevalence in women aged 15-49 > 0.5; ratio of prevalence in women aged 15-24 compared with men aged 15-24 is > 1.25; percentage of women giving birth with HIV in whom there is transmission to the child between 2% and 25%; the percent of people with HIV who are diagnosed is > 25%; of people with diagnosed HIV the proportion on ART > 68%; percentage of all people on ART who have viral load < 1000 copies/mL > 65%; percentage of women giving birth per year between 5% and 22%; proportion of women aged 15-49 who are sex workers > 0.2% and < 4%; proportion of people who have NNRTI resistance at ART initiation > 0. Model program (in which these variable names are used) available on figshare^

**Parameter name^**	**Description**	**Distribution sampled (value; % with value)**	**Motivation for distribution**
**Population demographics**			
inc_cat	Three future demographic structures with differing levels of population growth.	1: 33% 2: 33% 3: 33%	Different countries in sub-Saharan Africa have different population growth rates so we consider a range from around 1% to 3% per year (3)
Parameters relating to sexual behaviour			
base_rate_sw	Base rate per 3 months of a woman becoming a sex worker (also influenced by age and lifetime propensity)	0.001: 33% 0.0015: 33% 0.002: 33%	Informed by data on the proportion of women who are sex workers (4)
base_rate_stop_sexwork	Base rate per 3 months of a sex worker stopping sex work (also influenced by age)	0.03: 33% 0.015: 33% 0.01: 33%	Informed by data on the proportion of women who are sex workers and duration of sex work (4)
sex_beh_trans_matrix_m and sex_beh_trans_matrix_m	Matrix determining rate of transition between four levels of sexual behaviour. There are 15 versions for each of men and women.	1/15 probability for each transition matrix for men, the same for women	Due to the fact that data on sexual behaviour are from self-report, which is known to be highly unreliable, there is uncertainty over longitudinal patterns of sexual behaviour and the degree of skewness in the distribution of number of new partners we consider a range of possible matrices (15 for each gender = 225 possible combinations).
p_rred_p	Indicates the proportion of the population in whom the sexual risk behaviour is very low	0.1: 20% 0.3: 20% 0.5: 20% 0.7: 20% 0.9: 20%	In order to include a person-level effect on sexual behaviour this and the parameter below allow the population to be divided into three according to the lifelong tendency to have short term condomless sex partners.

**Parameter name^**	**Description**	**Distribution sampled (value; % with value)**	**Motivation for distribution**
p_hsb_p	Indicates the proportion of the population in whom the sexual risk behaviour has a tendency to be higher than average	0.05: 33% 0.1: 33% 0.2: 33%	As above
rred_a_p	Relative condomless sex levels by gender and age; four different patterns.	1: 25% 2: 25% 3: 25% 4: 25%	Uncertainty over levels of condomless sex by age so we consider a range of possibilities
eprate	Base rate of starting to have a long term condomless sex partner.	Lognormal(0.1, 0.25)	Informed by outputs that give the proportion of people with a long term condomless sex partner by age.
conc_ep	Parameter indicating the degree to which those with a long term condomless sex partner have a lower of higher probability of short term condomless sex partners than those without a long term condomless sex partner.	Lognormal(0,0.6)	This is likely to vary across setting scenarios and we wished to consider across the range. Again, this distribution of values was found, in certain (randomly selected) combination with other sexual behaviour parameter values to re-produce epidemics within the observed prevalence range.
ych_risk_beh_newp	Degree of reduction in condomless sex with short term partners per year from 1995 – 2000	0.90: 33% 0.85: 33% 0.80: 33%	In order to explain the decrease in incidence and prevalence of HIV in southern Africa in the late 1990s it is necessary to assume there was a reduction in condomless sex, which is supported by data in Zimbabwe (for example 5,6)
ych_risk_beh_ep	Degree of reduction in condomless sex per year with long term partners from 1995-2000	1: 20% 0.99: 20% 0.95: 20% 0.90: 20% 0.85: 20%	As above
ch_risk_diag_newp	Degree of reduction (fold change) in condomless sex with short term partners in a person diagnosed with HIV	0.7: 25% 0.8: 25% 0.75: 25% 1.00: 25%	Informed by (7)
ch_risk_diag	Degree of reduction in condomless sex with long term partner in a person diagnosed with HIV	0.7: 25% 0.8: 25% 0.75: 25% 1.00: 25%	As above
ych2_risk_beh_newp	Degree of change in condomless sex with short term partners per year from 2010 – 2015	1/0.96: 5% 1/0.99: 5% 1.0: 80% 0.99: 5% 0.96: 5%	It is uncertain whether there have been recent changes in condomless sex, hence a neutral distribution was used.
sex_age_mixing_matrix_m and sex_age_mixing_matrix_w	Matrix determining the age gender sexual mixing from male and female perspectives	6 different matrices for each gender persective, sampled at random	Uncertainty about mixing patterns by age and gender so we consider a range of these.
exp_setting_lower_p_vl1000	Whether there is exposure of individuals to settings with lower population viral suppression levels, due to migration (and return)	In 20% of runs	A subset of a population may have sexual exposure to others outside the population and the degree of this will be setting dependent so this is varied across model runs.

Parameter name^	Description	Distribution sampled (value; % with value)	Motivation for distribution
external_exp_factor<br><br>*rate_exp_set_lower_p_vl1000*	Measure of level of effect of the above exposure<br><br>Rate of exposure	Uniform(1,2)<br><br>Uniform(0, 0.01)	
higher_newp_with_lower_adhav	In people with lower adherence to ART there is a tendency for lower number of condomless partners	In 20% of runs	There could be correlation between ART adherence and sexual risk behaviour, in either direction
**Parameters relating to male circumcision**			
*circ_inc_rate_*	Determines the rate with which male circumcision increases over time	0.001: 20% 0002: 20% 0.003: 20% 0.01: 10% 0.03: 10% 0.05: 10% 0.07: 10%	This varies by country in the region (8).
**Parameters relating to being hard to reach for services**			
*p_hard_reach_w_*<br><br>*hard_reach_higher_in_men_ p*	Proportion of women that have a propensity to be hard to reach with prevention and testing servcices<br><br>The extent to which this is higher in men (in men this also includes propensity to be medically circumcised)	Uniform(0,0.05)<br><br>Uniform(0,0.1)	A small proportion of people have a long term propensity not to take up HIV services, for various possible reasons including stigma, physical barriers, etc. (9)
**Parameters relating to transmission**			
fold_change_w	The fold difference in female to male transmission rate compared with male to female, for a given viral load. Rate is higher in younger women.	1: 5% 1.25: 5% 1.5: 60% 2: 30%	Informed by the higher incidence and prevalence in women in younger age groups and some direct evidence. (10, 11)
Fold_change_sti	The fold difference in HIV acquisition risk for a person with a current STI.	Lognormal (3,0.3)	Multiple studies show a raised risk of acquisition but uncertainty over the effect size. (12)
fold_tr	A higher or lower risk of acquiring HIV for a given viral load in the partner	Lognormal (1, 0.3)	The convey uncertainty in the estimate of transmission rates
res_trans_factor_nn	Parameter affecting the probability that if NNRTI resistance mutation present in source partner that this is not present/detectable in virus new host	0.5: 20% 0.7: 20% 0.8: 20% 0.90: 20% 1.00: 20%	Informed by the values needed to lead to the range of transmitted NNRTI resistance observed (see Table 1 in paper)
res_trans_factor_ii	Parameter affecting the probability that if integrase inhibitor resistance mutation present in source partner that this is not present/detectable in virus new host	1: 80% 2: 20%	Little data available to inform this yet – we make the assumption in 80% of runs that transmission occurs.

Parameter name^	Description	Distribution sampled (value; % with value)	Motivation for distribution
super_infection	Whether we consider super-infection (which means a person with HIV can acquire HIV drug resistant HIV through a subsequent infection with a new viral strain).	Occurs in 50% of runs.	Super-infection can occur but its significance is uncertain but unlikely to be substantial. (13)
**Parameters relating to HIV testing**			
an_lin_incr_test	Parameter determining the rate of increase in HIV testing (any testing outside ANC)	0.00005: 25% 0.01: 25% 0.03: 25% 0.10: 25%	Range and pattern required to re-produce the observed range in proportion of HIV positive people diagnosed (see Table 1 of main paper).
*date_test_rate_plateau_*	Year in which the rate of HIV testing plateaus.	2011: 0.2 2013: 0.2 2015: 20% 2017: 20% 2018: 20%	Some countries have increased testing rates markedly and these have plateaued at different levels in different settings (e.g 14).
rate_testanc_inc	Rate of increase in testing in ANC clinics	0.03: 33% 0.05: 33% 0.1: 33%	Distribution is intended to reflect variation across setting scenarios. (14)
*incr_test_rate_sympt_*	The rate of increase per 3 months in the probability of a person with a WHO stage 3 or 4 disease is tested for HIV.	1.05: 20% 1.10: 20% 1.15: 20% 1.20: 20% 1.25: 20%	Little direct data on this parameter and wide range taken to reflect uncertainty and variation across settings.
max_freq_testing	A parameter defining the maximum frequency with which a person (non sex-worker) without AIDS or WHO stage 3 disease can test for HIV	Annually: 80% 6-monthly: 20%	Policy on frequency of testing varies by setting.
test_targeting	Parameter conveying the degree to which HIV testing is targetted towards people having condomless sex since last test.	Less targeting: 80% more targeting 20%	Data on condomless sex since last test not collected but likely to be a higher tendency to test if had sexual risks. We vary the degree of such “targeting”. Partially informed by outputs on testing yield.
**Parameters relating to pre-ART care and progression of HIV**			
fx	Multiplicative factor to alter the average rate of CD4 count decline in natural HIV progression (which thus alters the incubation period distribution).	0.7: 20% 0.85: 20% 1.0: 20% 1/0.85: 20% 1/0.7: 0.7	Derived based on consideration of evidence from natural history studies (15 – 19)
gx	Multiplicative factor allowing expression of uncertainty in rates of viral load increase over time in people untreated	1.0: 33% 1.5: 33% 2.0: 33%	There is uncertainty over rates of viral load increase (15)
prob_loss_at_diag<br><br>*prob_lossdiag_adctb*	Probability that a person is immediately lost after initial HIV diagnosis.<br><br>…if has an AIDS disease or TB at time of diagnosis	0.02: 25% 0.05: 15% 0.15: 30% 0.25: 25% 0.35: 5%<br><br>Beta(5,95)	e.g. (20)

Parameter name^	Description	Distribution sampled (value; % with value)	Motivation for distribution
prob_lossdiag_who3e	…if has an AIDS disease or TB at time of diagnosis	Beta(15,85)	
rate_lost	For people under care yet to start ART or previously have taken ART, the rate of being lost to care per 3 mths.	0.2: 33% 0.35: 33% 0.5: 33%	Uncertain and will vary by setting. Distribution chosen to reflect this. This is one of the parameters influencing the proportion of diagnosed people on ART.
rate_return	Probability of return to care for a person who has been diagnosed with HIV (and may have started ART) but is now lost and not on ART, without current WHO stage 3 or 4 disease, per 3 months.	0.05: 25% 0.10: 25% 0.25: 25% 0.5: 25%	As above
prob_return_adc	Probability of return to care for a person who has been diagnosed with HIV (and may have started ART) but is now lost and not on ART and has a WHO stage 4 condition. This is a probability that operates just for the 3-month period that the events occurs.	0.2: 5% 0.4: 15% 0.6: 30% 0.8: 50%	As above
rate_loss_persistence	Rate of loss from majority virus of transmitted resistance mutations (per 3 months)	0.00: 10% 0.005: 10% 0.01: 10% 0.015: 40% 0.02: 30%	(e.g. 21, 22)
**Parameters relating to people on ART**			
adh_pattern	Population adherence profile; described in terms of the proportion having a given average adherence and period-to-period variability in adherence. Note that adherence is additionally affected by age and gender.	A: 5% B: 5% C: 10% D: 5% E: 15% F: 15% G: 15% H: 15% I: 15%	Reflection of wide range of adherence profiles in different settings, informed by differences in proportions of people on ART with viral load suppression. This range leads to a range of levels of viral suppression and of resistance.

Parameter name^	Description	Distribution sampled (value; % with value)	Motivation for distribution
red_adh_tb_adc	Reduction in adherence to ART associated with currently having an AIDS defining condition / TB	logNormal(0.1, 0.5)	(23)
red_adh_tox_pop	The extent to which drug toxicity influences adherence to ART negatively.	red_adh_tox_pop_v = 0.05 50% 0.10 50% logNormal(red_adh_tox_pop_v, 0.5)	
add_eff_adh_nnrti	NNRTI drugs tend to have a longer half-life than PIs – this indicates the gain in effective adherence from NNRTIs due to this effect.	logNormal(0.10, 0.30)	
*red_adh_multi_pill_pop_*	The extent to which taking multiple ARVs separately tends to lead to lower adherence than a single once daily pill.	red_adh_multi_pill_pop_v = 0.05 50% 0.10 50% logNormal(red_adh_multi_pill_pop_v, 0.5)	
altered_adh_sec_line_pop	The extent of any increase in adherence in people switched to second line ART – this is in addition to any effects of enhanced adherence counselling after a measured viral load > 1000 copies/mL.	logNormal(0.05, 0.05)	
*sw_lower_art_adh_*	Whether sex workers (who are not engaged with a sex worker program, if it exists) tend to have a lower adherence to ART, due to poorer access and mobility.	No: 80% yes: 20%	
pr_art_init	Probability of ART initiation per 3 months in a person in care who is eligible according to current criteria.	0.4: 25% 0.5: 25% 0.6: 25% 0.7: 25%	These parameters contribute to determine the proportion of HIV diagnosed people who are on ART. The distributions are chosen such that combinations of these parameters lead to observed proportions of HIV diagnosed people on ART (e.g. Population Health Impact Surveys (24)
prob_lost_art	For a person who interrupts / stops ART the probability that they are simultaneously lost from care.	0.5: 25% 0.6: 20% 0.7: 20% 0.8: 20% 0.9: 20%	(e.g. 25)
rate_restart	Rate of restart of ART for people who previously have been on ART and have returned to care, per 3 months.	0.2: 25% 0.4: 25% 0.6: 25% 0.8: 25%	Assumed to be high, given the person has returned to care. Most people who are regularly seen in clinics who have previously started ART are on ART.
rate_int_choice	Rate of interruption / stopping of ART per 3 months. Also influenced by current drug toxicity and underlying tendency to adhere.	0.001: 20% 0.005: 10% 0.01: 10% 0.015: 20% 0.02: 20% 0.03: 20%	(25)

Parameter name^	Description	Distribution sampled (value; % with value)	Motivation for distribution
incr_rate_int_low_adh	Parameter indicating the extent to which people with a long-term average adherence in the lowest group have a multiplicatively increased risk of ART interruption.<br><br>Effect of current low adherence on risk of treatment interruption / discontinuation.	1: 50% 2: 25% 5: 25%	(26)
pr_switch_line	Probability of switch to second line per 3 months in a person who has fulfilled the failure criteria for first line failure.	0.20: 50% 0.50: 50%	In several settings, including Zimbabwe, the proportion of people who have started second line ART is consistent with a value for *pr_switch*_line of below 0.1 (e.g. Lesotho, Malawi) (14, 27, 28)
clinic_not_aw_int_frac	If a person interrupts ART, the probability that this is not disclosed to the clinic and they are classified as being on ART	0.1: 20% 0.3: 20% 0.5: 20% 0.7: 20% 0.9: 20%	Uncertain and will vary by setting, hence a broad distribution.
fold_change_mut_risk	Fold difference in rate of accumulation of mutations (for all drugs) compared with base case.	1: 80% 2: 10% 0.5: 10%	To consider that the rate of resistance mutation acquisition is higher or lower than the rate assumed, reflecting some uncertainty. This relates to all resistance mutations.
*rate_res_ten_*	Parameter reflecting the rate of acquisition of tenofovir resistance. The value of 0.1 was derived based on European cohort data and the value of 0.3 reflects the potentially higher value for subtype C in southern Africa.	0.1: 33% 0.2: 33% 0.3: 33%	(29)
poorer_cd4_rise_on_fail_nn<br><br>*poorer_cd4_rise_on_fail_ii*	This indicates the extent of poorer CD4 rise per 3 months on failing NNRTI based regimens (compared with PI)<br><br>This indicates whether the poorer CD4 rise also applied to failing INSTI based regimens	Normal(-6, 3)<br><br>no: 50% yes: 50%	(30)
adh_effect_of_meas_alert	The effect of having a viral load measured > 1000 copies/mL on adherence, due to the enhanced adherence intervention.	0.7:70% 0.35: 15% 0.9: 15%	Uncertainty over the effect size.
prob_vl_meas_done	Probability of a viral loead measure being done. This probability operates for each time a viral load is due to be tested.	0.1: 33% 0.7: 33% 1.00: 33%	Variation in viral load implementation in different settings. Note that in half of settings with value 0 there is CD4 count monitoring of people on ART in place.
switch_for_tox	Whether the ART program manages to implement drug substitutions in response to specific toxicities experienced by patients.	No: 80% Yes: 20%	This will vary by program but generally not widespread.

Parameter name^	Description	Distribution sampled (value; % with value)	Motivation for distribution
zero_3tc_activity_m184	activity of 3TC in presence of M184V mutation	No: 80% Yes: 20%	To consider alternative assumptions; distribution broadly reflects the uncertainty.
zero_ten_activity_k65	activity of 3TC in presence of K65R mutation	No: 80% Yes: 20%	To consider alternative assumptions; distribution broadly reflects the uncertainty.
higher_rate_res_dol	Whether there is a higher rate of resistance to dolutegravir than the base assumption (i.e. 4 times lower than efavirenz compared with 13 times lower in base case).	No: 90% Yes: 10%	To consider alternative assumptions; distribution broadly reflects the uncertainty.
rel_rate_res_cla_dol	Parameter indicating the extent of greater resistance risk in people on cabotegravir compared with dolutegravir	2.0 fold: 90% 1.5 fold: 10%	(31)
dol_efa_cla_rla_potency	The level of potency assigned to dolutegravir, efavirenz, cabotegravir and rilpivirine (all are assumed to have the same potency), where potency of PI/r is 2 and NRTIs is 1.	1.5: 50% 2.0: 50%	Previously we considered higher values for potency of dolutegravir compared with efavirenz [120-140]. In the light of results from NAMSAL and ADVANCE we modified so that both drugs have a potency 1.5. (32, 33)
cla_time_to_lower_threshold_g	Paremeter indicating distribution of time over which cabotegravir drug levels remain such that there is a risk of resistance mutations developing (see Methods in main paper).	Group 1: 33% Group 2: 33% Group 3: 33%	(34)
*rel_onart_la_drugs_*	Rate of ARV interruption / restart with iCTG/RPV compared with oral ARVs.	0.5: 33% 1.0: 33% 2.0: 33%	Uncertain in practice as yet as not rolled out.
*prop_bmi_ge23_*	Proportion of people initiating dolutegravir who have BMI $\geq$ 23	0.5: 50% 0.75: 50%	uncertainty over the proportion of the population starting dolutegravir who have BMI > 23 and hence a possible negative effect of weight gain on dolutegravir. (35, 36)
*incr_mort_risk_dol_weightg_*	Rate ratio for mortality in people on dolutegravir who had BMI $\geq$ 23 at start, due to dolutegravir induced weight gain.	1: 1% 1.1: 16% 2: 17% 2.1: 17% 2.2: 17% 3.0: 17% 4.0: 15%	Wide distribution within plausible bounds reflecting uncertainty (37 – 40)
nnrti_res_no_effect	Effect of NNRTI resistance mutations on activity of efavirenz (base case: K103N 0 activity, G190A 0.25 activity, Y181C 0.25 activity)	0.25: 25% 0.5: 5% 0: 75%	Some uncertainty over this. With this distribution the average odds ratio for VL > 1000 at 1 year from start of ART associated with pre-treatment NNRTI drug resistance = 3.3 (compared with 3.9 in a recent meta-analysis (41)
tox_weightg_dol	Whether weight gain is treated as a toxicity that has an associated increased risk of ART interruption	no: 50% yes: 50%	Weight gain does not seem to be mentioned as troublesome to people on dolutegravir, but this could change with time.

Parameter name^	Description	Distribution sampled (value; % with value)	Motivation for distribution
double_rate_gas_tox_taz	Parameter related to the rate of gastrointestinal toxity relating to atazanavir. Whether base rate is doubled or not.	Yes: 50% No: 50%	Uncertainty over gastrointestinal toxity relating to atazanavir – consider possibility that this has been underestimated.
rr_int_tox	Increased rate of ART interruption according to presence of a drug toxicity.	2-fold: 40% 10-fold: 60%	Consider possibiity that ART interruption is substantially more highly related to drug toxicity than base case
greater_disability_tox	Parameter to allow consideration of a greater disability weight associated with drug toxicity (0.25) compared with the base assumption (of 0.05)	No: 90% Yes: 10%	To reflect the uncertainty and perceived relatively low likelihood that value is as high as 0.25.
*greater_tox_zdv_*	Whether the toxicity associated with zidovudine is greater than the base assumption.	No: 50% Yes: 50%	(42)
**Parameter relating to pregnancy**			
prob_pregnancy_base	Parameter determining base rate of pregnancy for women having condomless sex (to which there is an effect of age)	Uniform (0.03, 0.11)<br>if inc_cat = 1 then prob_pregnancy_base increased 1.75-fold<br>if inc_cat = 3 then prob_pregnancy_base decreased 1.75 -fold	Variability between settings in fertility rate.
rate_birth_with_infected_child	Parameter determining the risk of mother to child transmission (MTCT), for a given level of mother viral load.	0.3: 5% 0.4: 25% 0.5: 60% 0.6: 10%	To produce plausible variation in the MTCT rate.
*oth_dol_adv_birth_e_risk_*	Risk of dolutegravir-induced adverse birth event, due to dolutegravir-induced weight gain	0.0005: 20% 0.0015: 40% 0.002: 20% 0.003: 20%	Wide distribution within plausible bounds reflecting uncertainty. (43)

^ model program (in which these variable names are used) available on figshare (see main paper)

Table S2. Disability weights

Values are 1 in each three-month period except for the following:

Condition in current 3-month period	Disability weight for current 3-month period	Source
Any drug toxicity in current 3-month period<br><br>Any WHO stage 3 condition (except TB) in current 3-month period<br><br>TB in current 3-month period<br><br>Any WHO stage 4 condition in current 3-month period	0.05<br><br>0.22<br><br>0.40<br><br>0.54	(44)

Table S3. Unit Costs

Item	Unit Cost	Source / explanation
Drug costs per year:<br><br>TLE<br><br>TLD<br><br>ZL-PI (PI atazanavir)<br><br>ZLD	 <br>$78 ($65 without supply chain costs<br><br>$78 ($65 without supply chain costs)<br><br>$318 ($265 without supply chain costs)<br><br>$126 ($105 without supply chain costs)	(45)
Cost of treatment of a WHO stage 4 condition over 3 months (cost is incurred for 3 months)<br><br>Cost of treatment of a WHO stage 3 condition over 3 months (cost is incurred for 3 months)<br><br>Cost of treatment of TB per 3 months (cost is incurred for 6 months)<br><br>Cotrimoxazole annual cost	$200<br><br>$20<br><br>$50<br><br>$5	Specific data not available on average unit costs of treating WHO stage 3 and 4 conditions and per clinic visit costs - costs used are informed by evidence synthesis from studies that cost according to current CD4 count of those in pre-ART care, cost of ART initiation, which also include costs of CD4 tests (46)
CD4 count measurement	$10	47, 48
Viral load measurement:	$22	Human resource costs $3, sample collection consumables $2, relaying of results $2 (this costing information was provided by Médecins Sans Frontierès (MSF) (including equipment and other costs such as consumables, maintenance and shipping) $15. Updates are consistent with this cost (49)
Non-ART programme costs per year, $40 per year if on tiered care due to viral load < 1000	$80	Bill and Melinda Gates Foundation tiered care meeting report (the per client cost of running the Khayelitsha adherence clubs was $58 per client per year compared to standard clinic care of $108 per client per year. At the Infectious Disease Institute in Kampala, the annual costs per client for physician, nurse, and pharmacy only visits were $60, $45, and $19, respectively) (50 – 52)

Item	Unit Cost	Source / explanation
Cost of the targeted adherence counselling intervention triggered by a viral load > 1000 copies/mL	$10	Assumption
HIV test (including personnel costs)	$3.70	Personal communication. CHAI.
Annual cost of treatment for a child born with HIV	$160	This cost was estimated based on a drug cost of $75 per year, a one-off cost of early infant diagnosis of $22, cost of viral load testing of $22 per year, costs of clinic visits of $40 or $80 per year (depending on whether viral load is suppressed), assuming 50% of children will achieve viral suppression. This is likely to be a lower limit cost per year.

References

1. Phillips AN, Bansi-Matharu L, Venter F, Havlir D, Pozniak A, Kuritzkes D, et al. Updated assessment of risks and benefits of dolutegravir versus efavirenz in new antiretroviral treatment initiators in sub-Saharan Africa: modelling to inform treatment guidelines. Lancet HIV 2020; 7: E193-E200.
2. Phillips AN, Cambiano V, Johnson L, et al. Potential Impact and Cost-Effectiveness of Condomless-Sex–Concentrated PrEP in KwaZulu-Natal Accounting for Drug Resistance. J Infect Dis 2019. https://doi.org/10.1093/infdis/jiz667
3. (https://population.un.org/wpp/)
4. Vandepitte J, Lyerla J, Dallabetta G, Crabbe F, Alary M, Buve A. Estimates of the number of female sex workers in different regions of the world. Sex Transm Infect 2006;82(Suppl III):iii18–iii25. doi: 10.1136/sti.2006.020081
5. Gregson S, Gonese E, Hallett TB, et al. HIV decline in Zimbabwe due to reductions in risky sex? Evidence from a comprehensive epidemiological review. Int J Epid 2010;39:1311–1323.
6. Halperin DT, Mugurungi O, Hallett TB, Muchini B, Campbell B, et al. (2011). A Surprising Prevention Success: Why Did the HIV Epidemic Decline in Zimbabwe? PLoS Med 8(2): e1000414. doi:10.1371/journal.pmed.1000414
7. Fonner VA, Denison J, Kennedy CE, O'Reilly K, Sweat M. Voluntary counseling and testing (VCT) for changing HIV-related risk behavior in developing countries. Cochrane Database of Systematic Reviews 2012, Issue 9. Art. No.: CD001224. DOI:10.1002/14651858.CD001224.pub4.
8. Cork MA, Wilson KF, Perkins S, Collison ML, Deshpande A et al. Mapping male circumcision for HIV prevention efforts in sub-Saharan Africa. BMC Medicine (2020) 18:189. https://doi.org/10.1186/s12916-020-01635-5
9. Grimsrud A, Wilkinson L, Eshun-Wilson I, Holmes C, Sikazwe6 I, Katz IT. Understanding Engagement in HIV Programmes: How Health Services Can Adapt to Ensure No One Is Left Behind. Current HIV/AIDS Reports (2020) 17:458–466
10. Masson L, Passmore J, Liebenberg LJ, Werner L, Baxter C, Arnold KB. Genital Inflammation and the Risk of HIV Acquisition in Women. Clinical Infectious Diseases 2015;61(2):260–9
11. Nicolosi, A. et al. The Efficiency of Male-to Female and Female-to-Male Sexual Transmission of the Human Immunodeficiency Virus: A Study of 730 Stable Couples. Epidemiology 5, 570-575 (1994).
12. Cohen, M. S. Sexually transmitted diseases enhance HIV transmission: no longer a hypothesis. The Lancet 351, S5-S7 (1998).
13. Smith, D. M. et al. HIV drug resistance acquired through superinfection. AIDS 19, 1251-1256 (2005).
14. Government of Malawi Ministry of Health Quarterly Reports.
15. Pantazis N, Touloumi G. Bivariate modelling of longitudinal measurements of two human immunodeficiency type 1 disease progression markers in the presence of informative drop-outs. JRSS C 2005; 54: 405-423.
16. Sabin CA, Devereux H, Phillips AN, et al. Course of viral load throughout HIV-1 infection. JAIDS 2000; 23:172-177.
17. Hubert J-B, Burgard M, Dussaix E, et al. Natural history of serum HIV-1 RNA levels in 330 patients with known date of infection. AIDS 2000; 14:123-131.
18. O'Brien TR, Rosenberg PS, Yellin F, et al. Longitudinal HIV-1 RNA levels in a cohort of homosexual men. JAIDS 1998; 18:155-161.
19. Henrard DR, Phillips JF, Muenz LR et al. Natural history of HIV-1 cell-free viraemia. JAMA 1995; 274: 554-558.
20. Rosen S, Fox MP. Retention in HIV Care between Testing and Treatment in Sub-Saharan Africa: A Systematic Review. PLOS Medicine 2011; 8. Article Number: e1001056

21. Jain V, Sucupira MC, Bacchetti P, Hartogensis W, Diaz RS, Kallas EG, et al. Differential Persistence of Transmitted HIV-1 Drug Resistance Mutation Classes. J Infect Dis 2011; 203(8):1174-1181.
22. Yang W-L, Kouyos RD, Böni J, Yerly S, Klimkait T, Aubert V, et al. Persistence of Transmitted HIV-1 Drug Resistance Mutations Associated with Fitness Costs and Viral Genetic Backgrounds. PLoS Pathog 2015 11(3): e1004722. doi:10.1371/journal.ppat.1004722
23. Heestermans T, Browne JL, Aitken SC, et al. Determinants of adherence to antiretroviral therapy among HIV-positive adults in sub-Saharan Africa: a systematic review. BMJ Global Health 2016;1:e000125. doi:10.1136/bmjgh-2016-000125
24. Population Health Impact Surveys. https://phia.icap.columbia.edu/
25. McMahon JH, Spelman T, Ford N, Greig J, Mesic A, Ssonko C et al. Risk factors for unstructured treatment interruptions and association with survival in low to middle income countries. AIDS Res and Therapy 2016; 13. Article Number: 25
26. Agbaji OO, Abah IO, Falang KD, Ebonyi AO, Musa J, Ugoagwu P, et al. Treatment Discontinuation in Adult HIV-Infected Patients on First-Line Antiretroviral Therapy in Nigeria. Curr HIV Research 2015; 13: 184-192 DOI: 10.2174/1570162X1303150506181945
27. Fox MP et al. Rates and Predictors of Failure of First-line Antiretroviral Therapy and Switch to Second-line ART in South Africa. JAIDS 2012; 60:428–437
28. Johnston, V., Fielding, K.L., Charalambous, S., Churchyard, G., Phillips, A., & Grant, A.D. 2012. Outcomes following virological failure and predictors of switching to second-line antiretroviral therapy in a South African treatment program. J.Acquir.Immune.Defic.Syndr., 61, (3) 370-380 available from: PM:22820803
29. Tenores Study Group. Global epidemiology of drug resistance after failure of WHO recommended first-line regimens for adult HIV-1 infection: a multicentre retrospective cohort study. Lancet Infect Dis 2016. http://dx.doi.org/10.1016/S1473-3099(15)00536-8
30. Ledergerber, B., Lundgren, J.D., Walker, A.S., Sabin, C., Justice, A., Reiss, P., Mussini, C., Wit, F., d'Arminio, M.A., Weber, R., Fusco, G., Staszewski, S., Law, M., Hogg, R., Lampe, F., Gill, M.J., Castelli, F., & Phillips, A.N. 2004. Predictors of trend in CD4-positive T-cell count and mortality among HIV-1-infected individuals with virological failure to all three antiretroviral-drug classes. Lancet, 364, (9428) 51-62 available from: PM:15234856.
31. Oliveira R-I, Anstett K, Mésplède T, Routy J-P, Robbins M-A, Bluma GB and the Montreal Primary HIV (PHI) Cohort Study Group. Selective resistance profiles emerging in patient-derived clinical isolates with cabotegravir, bictegravir, dolutegravir, and elvitegravir. Retrovirology 2018 15:56. https://doi.org/10.1186/s12977-018-0440-3
32. The NAMSAL ANRS 12313 Study Group. Dolutegravir-based or low-dose efavirenz-based regimen for the treatment of HIV-1. N. Engl. J. Med. 381, 816–826 (2019).
33. Venter WDF, Moorhouse M, Sokhela S, et al. Dolutegravir plus two different prodrugs of tenofovir to treat HIV. N Engl J Med 2019; 381:803–15.
34. https://www.clintonhealthaccess.org/the-state-of-the-hiv-market-in-low-and-middle-income-countries-2/. HIV MARKET REPORT The state of HIV treatment, testing, and prevention in low- and middle-income countries Issue 10, September 2019.
35. Msyamboza KP, Ngwira B, Dzowela T, Mvula C, Kathyola D, et al. (2011) The Burden of Selected Chronic Non-Communicable Diseases and Their Risk Factors in Malawi: Nationwide STEPS Survey. PLoS ONE 6(5): e20316. doi:10.1371/journal.pone.0020316
36. Thorogood M, Connor M, Tollman S, Lewando Hundt G, Fowkes G, Marsh J. A cross-sectional study of vascular risk factors in a rural South African population: data from the Southern African Stroke Prevention Initiative (SASPI). BMC Public Health 2007, 7:326. doi:10.1186/1471-2458-7-326

37. Berrington de Gonzalez A, Hartge P, Cerhan JR, Flint AJ, Hannan L, MacInnis RJ. Body-Mass Index and Mortality among 1.46 Million White Adults. N Engl J Med. 2010 December 2; 363(23): 2211–2219. doi:10.1056/NEJMoa1000367.
38. Flegal KM, Kit BK, Orpana H, Graubard BI. Association of All-Cause Mortality With Overweight and Obesity Using Standard Body Mass Index Categories: A Systematic Review and Meta-analysis. JAMA. 2013 January 2; 309(1): 71–82. doi:10.1001/jama.2012.113905.
39. Achhra AC, Sabin CA, Ryom L, Hatleberg C, d'Aminio Monforte, de Wit S, et al. Body Mass Index and the Risk of Serious Non-AIDS Events and All-Cause Mortality in Treated HIV-Positive individuals: D:A:D Cohort Analysis. J Acquir Immune Defic Syndr _ Volume 78, Number 5, August 15, 2018.
40. Kivimäki M, Kuosma , Ferrie JF, Luukkonen R, Nyberg ST, Alfredsson L, et al. Overweight, obesity, and risk of cardiometabolic multimorbidity: pooled analysis of individual-level data for 120 813 adults from 16 cohort studies from the USA and Europe. Lancet Public Health 2017; 2: e277–85.
41. Kanters S, et al. Meta-analysis of association between baseline NNRTI resistance and virologic failure prepared for WHO guidelines meeting June 2019.
42. https://clinicalinfo.hiv.gov/en/guidelines/pediatric-arv/zidovudine
43. Cresswell J, Campbell OMR, De Silva MJ, Filippi V. Effect of maternal obesity on neonatal death in sub-Saharan Africa: multivariable analysis of 27 national datasets. Lancet 2012; 380: 1325–30
44. Salomon JA, Vos T, Hogan DR, et al. Common values in assessing health outcomes from disease and injury: disability weights measurement study for the Global Burden of Disease Study 2010. Lancet 2012; 380: 2129–43.
45. CHAI Market report 2020. clintonhealthaccess.org/hiv-mid-year-market-memo-2020/
46. Eaton J et al. Health benefits, costs, and cost-effectiveness of earlier eligibility for adult antiretroviral therapy and expanded treatment coverage: a combined analysis of 12 mathematical models. Lancet Global Health 2014: E23-E34
47. Hyle, E. P., Jani, I. V, Lehe, J., Su, A. E., Wood, R., Quevedo, J., … Walensky, R. P. (2014). The Clinical and Economic Impact of Point-of-Care CD4 Testing in Mozambique and Other Resource-Limited Settings: A Cost-Effectiveness Analysis. PLoS Med, 11(9), e1001725. doi:10.1371/journal.pmed.1001725.
48. Keebler D, Revill P, et al. How Should HIV Programmes Monitor Adults on ART? A Combined Analysis of Three Mathematical Models. Lancet Global Health 2014. E35-E43.
49. Global Fund Releases. http://www.theglobalfund.org/en/mediacenter/newsreleases/2015-06-10_New_Approach_on_HIV_Viral_Load_Testing/ http://www.theglobalfund.org/en/procurement/viral-load-early-infant-diagnostics/)
50. Siapka M, Remme M, Dayo Obure C, Maier C, Dehne KL, Vassall A. Is there scope for cost savings and efficiency gains in HIV services? A systematic review of the evidence from low- and middle-income countries. Bull World Health Organ 2014;92:499–511AD doi:http://dx.doi.org/10.2471/BLT.13.127639;
51. Tagar E, Sundaram M, Condliffe K, Matatiyo B, Chimbwandira F, et al. Multi-Country Analysis of Treatment Costs for HIV/AIDS (MATCH): Facility-Level ART Unit Cost Analysis in Ethiopia, Malawi, Rwanda, South Africa and Zambia. PLoS ONE 2014; 9(11): e108304. doi:10.1371/journal.pone.0108304;
52. Menzies NA, Berruti AA, Blandford JM (2012) The Determinants of HIV Treatment Costs in Resource Limited Settings. PLoS ONE 7(11): e48726. doi:10.1371/journal.pone.0048726
